# Supplementary material for: Histone chaperone ASF1 mediates H3.3-H4 deposition in Arabidopsis
Source: Nat Commun. 2022 Nov 15;13:6970. doi: 10.1038/s41467-022-34648-0 (PMC9666630; doi:10.1038/s41467-022-34648-0)
Supplement: Supplementary file 1 — Supplementary Information [file 41467_2022_34648_MOESM1_ESM.pdf]

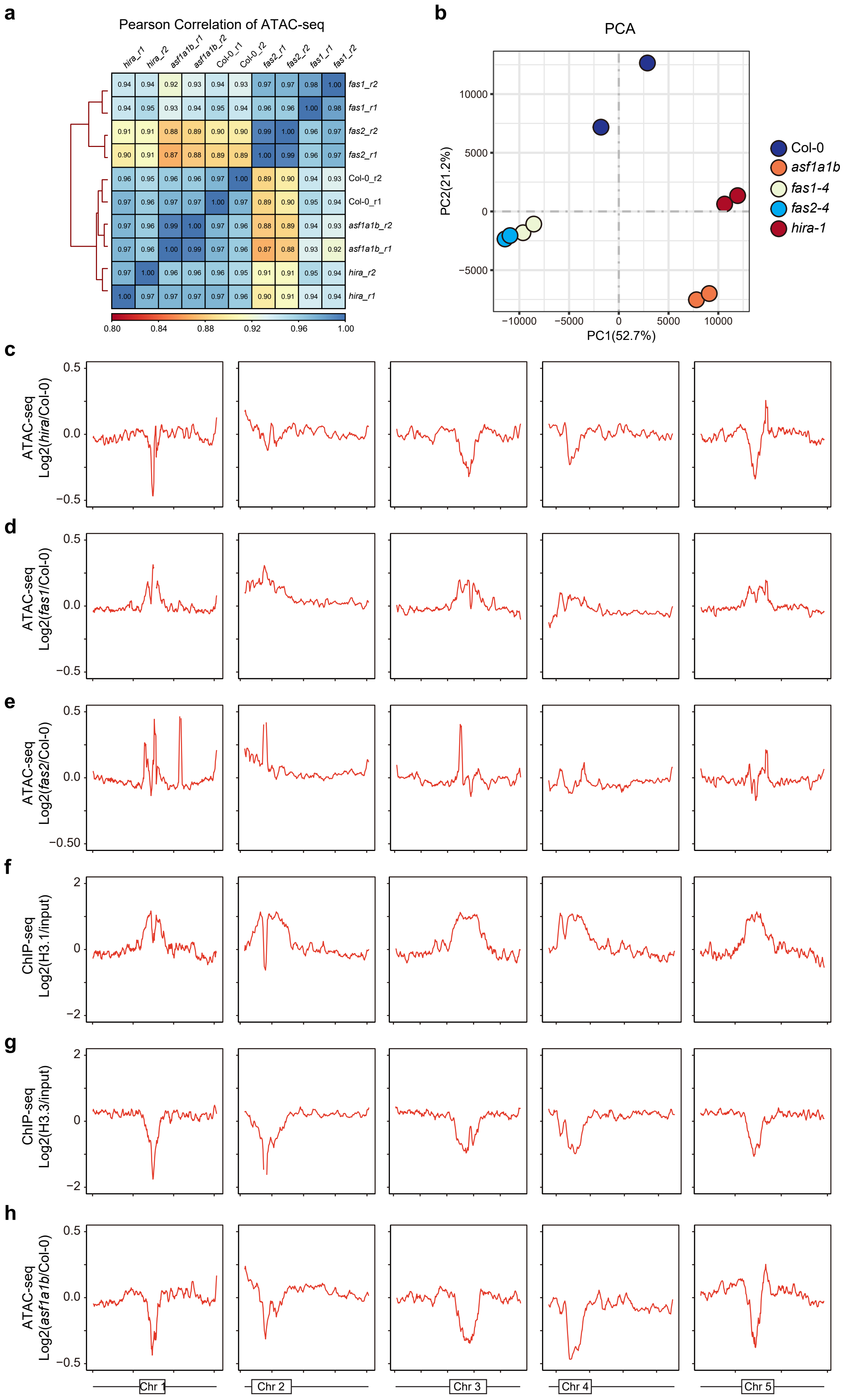

a
Pearson Correlation of ATAC-seq
hira_r1
hira_r2
asf1a1b_r1
asf1a1b_r2
Col-0_r1
Col-0_r2
fas2_r1
fas2_r2
fas1_r1
fas1_r2
0.80
0.84
0.88
0.92
0.96
1.00
b
PCA
PC1(52.7%)
PC2(21.2%)
Col-0
asf1a1b
fas1-4
fas2-4
hira-1
c
ATAC-seq
Log2(hira/Col-0)
d
ATAC-seq
Log2(fas1/Col-0)
e
ATAC-seq
Log2(fas2/Col-0)
f
ChIP-seq
Log2(H3.1/input)
g
ChIP-seq
Log2(H3.3/input)
h
ATAC-seq
Log2(asf1a1b/Col-0)
Chr 1
Chr 2
Chr 3
Chr 4
Chr 5

Figure S1. Comparison of ATAC-seq and H3s distribution.
(a) Correlation coefficient between replicates of Col-0, asf1a1b, hira-1, fas1-4, and fas2-4 ATAC-seq data. Pearson method was used to calculate correlation coefficient.
(b) Principal component analysis (PCA) from replicates of Col-0, asf1a1b, hira-1, fas1-4, and fas2-4 ATAC-seq data.
Differential (mutant vs Col-0) ATAC-seq signals plotted over Arabidopsis chromosomes in hira-1 **(c)**, fas1-4 **(d)**, fas2-4 **(e)** and asf1a1b **(h)**. H3.1 **(f)** and H3.3 **(g)** ChIP signals in Col-0 over Arabidopsis chromosomes. Reads per million mapping reads (RPKM) is calculated in 100-kb bins and normalized with Col-0 or ChIP input. Boxes correspond to the heterochromatic region.

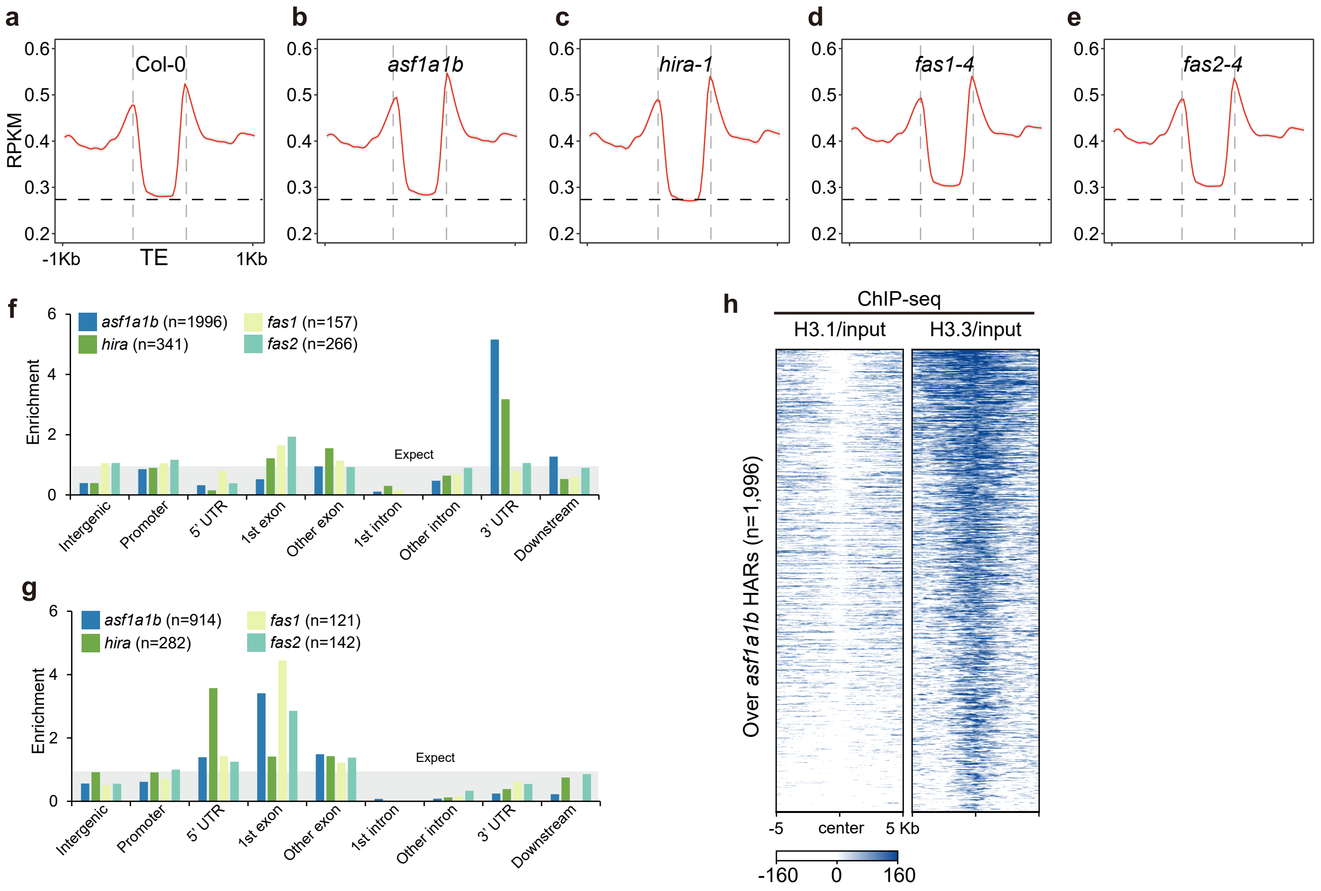

Figure S2. Distribution of ATAC-seq reads over transposon elements (TE) and genomic distribution of the differentially enriched ATAC-seq peaks.

Metaplot of Col-0 **(a)**, asf1a1b **(b)**, hira-1 **(c)**, fas1-4 **(d)**, and fas2-4 **(e)** ATAC-seq data over TE (n=32153) including 1 Kb flanking sequence.

(f) Genomic distribution of asf1a1b, hira-1, fas1-4, and fas2-4. Highly Accessible Regions (HARs) represent genomic regions where mutants have higher chromatin accessibility than wild-type.

(g) Genomic distribution of asf1a1b, hira-1, fas1-4, and fas2-4. Lowly Accessible Regions (LARs) represent genomic regions where mutants have lower chromatin accessibility than wild-type ones. Enrichment = (number of sample peaks)/(number of randomly selected peaks).

(h) Heatmap showing ChIP-seq signals of H3.1/input, H3.3/input over asf1a1b HARs (n=1,996).

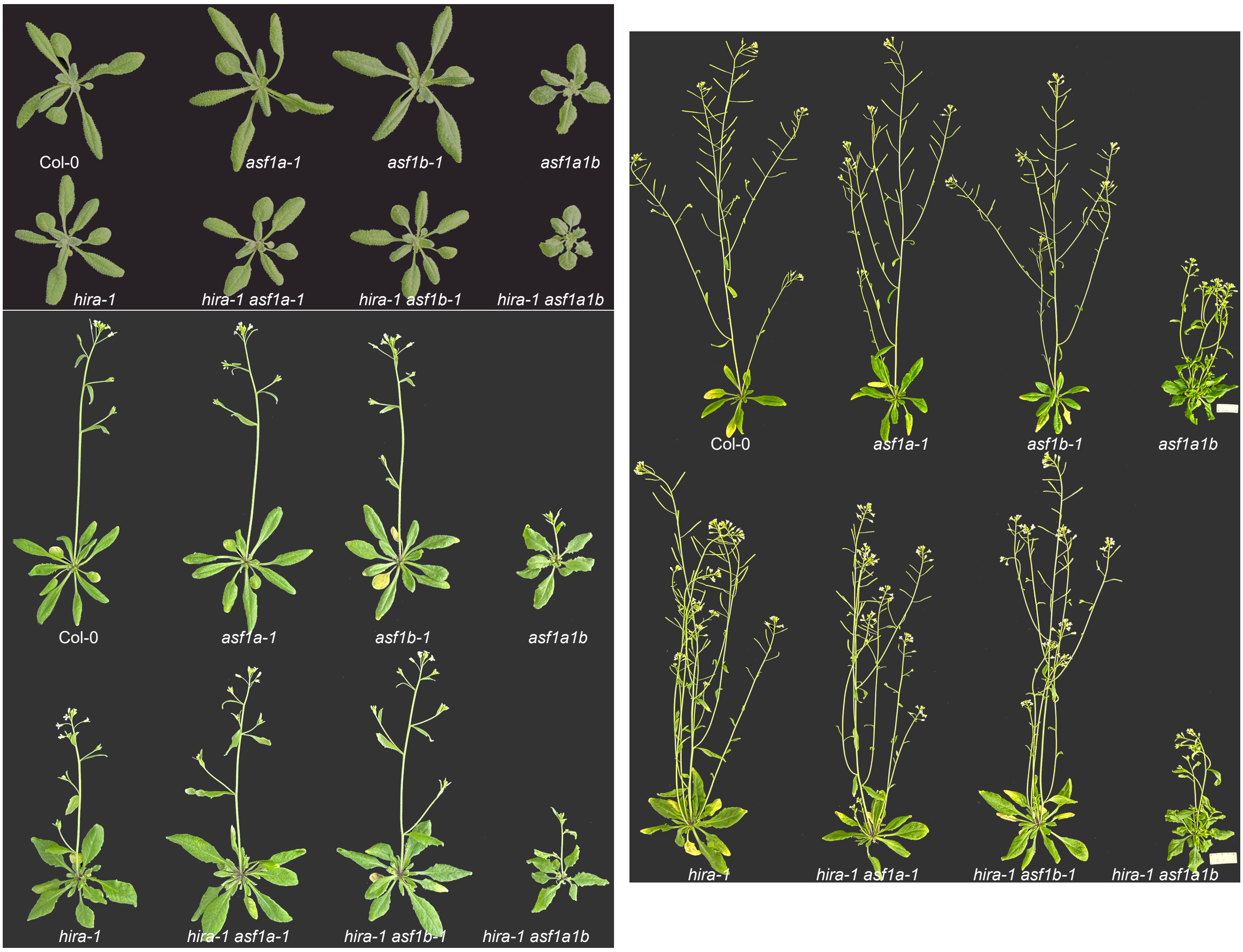

Figure S3. Morphology of Col-0, asf1a-1, asf1b-1, hira-1, asf1a1b, hira-1 asf1a-1, hira-1 asf1b-1, and hira-1 asf1a1b.

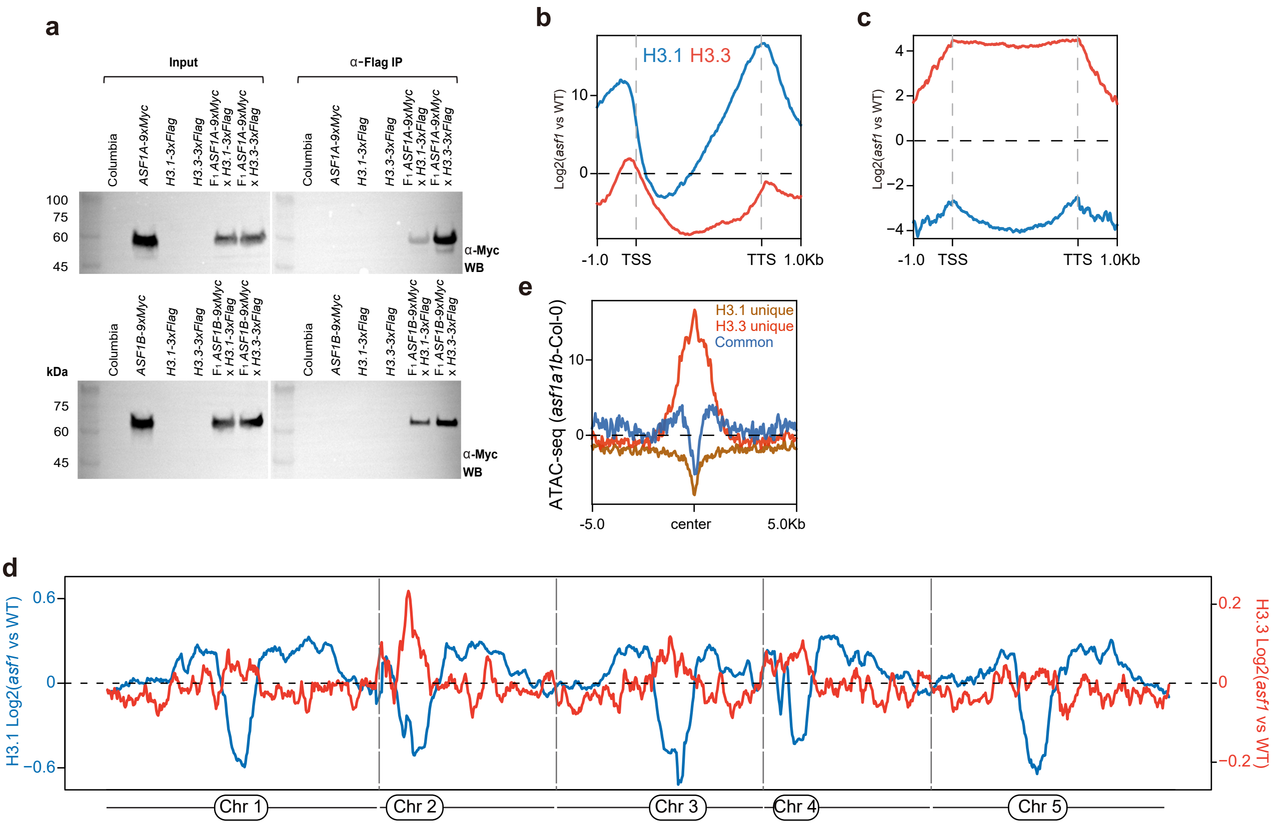

Figure S4. ASF1 mutation causes a redistribution of H3.3 and H3.1.
(a) Co-immunoprecipitation assay of ASF1A, ASF1B, H3.1, and H3.3. Labels to the right indicate the antibody used for the western blot.
(b) Metaplot of H3.1 and H3.3 variation (asf1a1b vs. Col-0) over the gene body and 1 Kb flanking sequence of genes with coding regions lengths over 150bp (n=26816). The Blue and the red line represents H3.1 and H3.3 variation, respectively.
(c) Metaplot of H3.1 and H3.3 variation (asf1a1b vs Col-0) over TE (n=32153) including 1 Kb flanking sequence. The Blue and the red line represents H3.1 and H3.3 variation, respectively.
(d) Metaplot of H3.1 and H3.3 variation (asf1a1b vs Col-0) over 5 chromosomes splitting into 100 Kb bins. The Blue and the red line represents H3.1 and H3.3 variation, respectively.
(e) Variation of ATAC-seq (asf1a1b vs Col-0) over H3.1-unique (n=24841), H3.3-unique (n=28629), and H3.1/H3.3 common (n=15051) peaks.

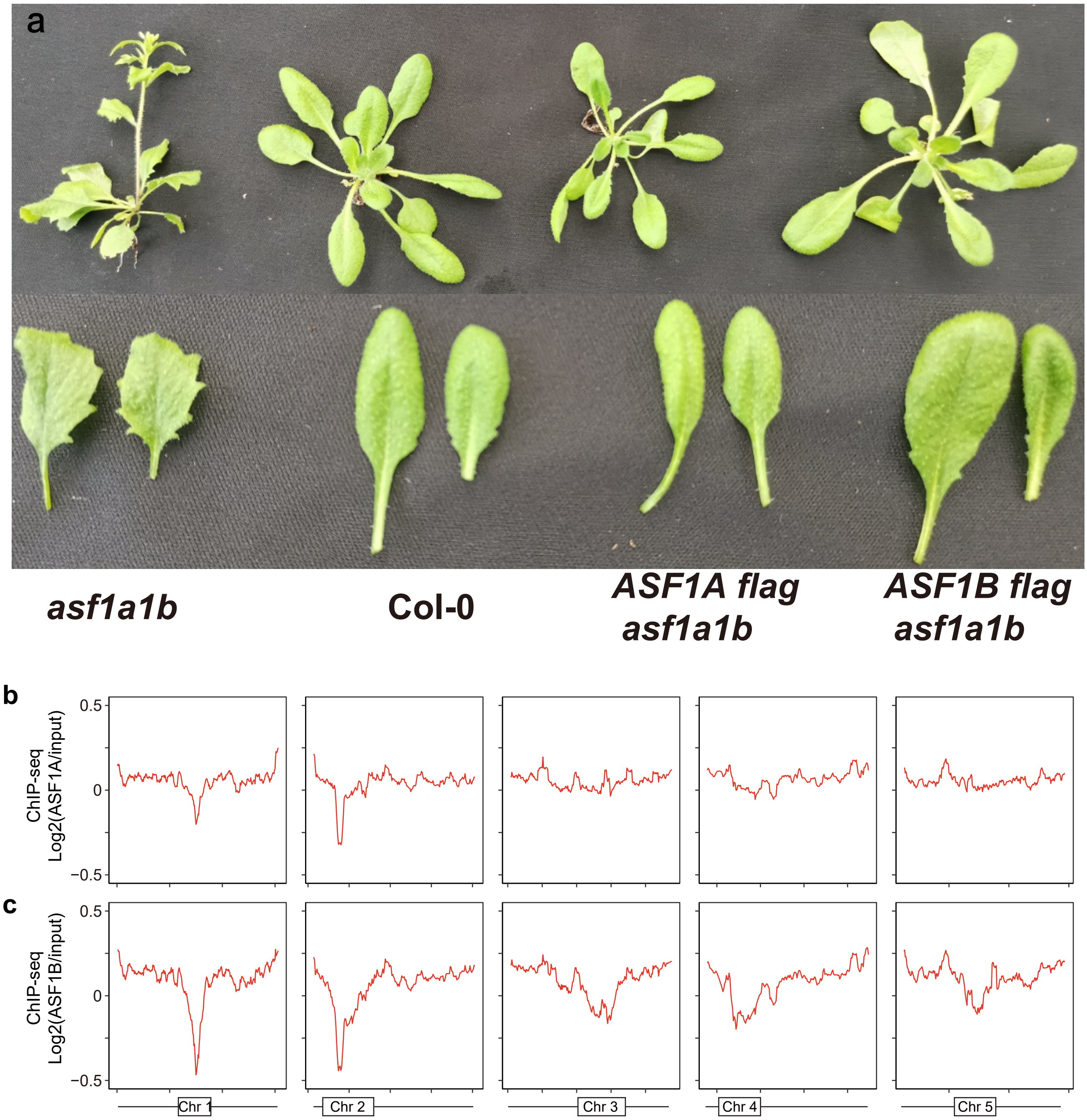

Figure S5. Genomic distribution of ASF1 proteins.
(a) Morphological phenotype of asf1a1b mutants transformed with ASF1A-Flag and ASF1B-Flag.
(b-c) Whole-genome distribution of ASF1A and ASF1B ChIP-seq signal over Arabidopsis chromosomes. Reads per million mapping reads (RPKM) were calculated in 100-Kb bins and normalized with Col-0 or ChIP input. Boxes correspond to the heterochromatic regions.

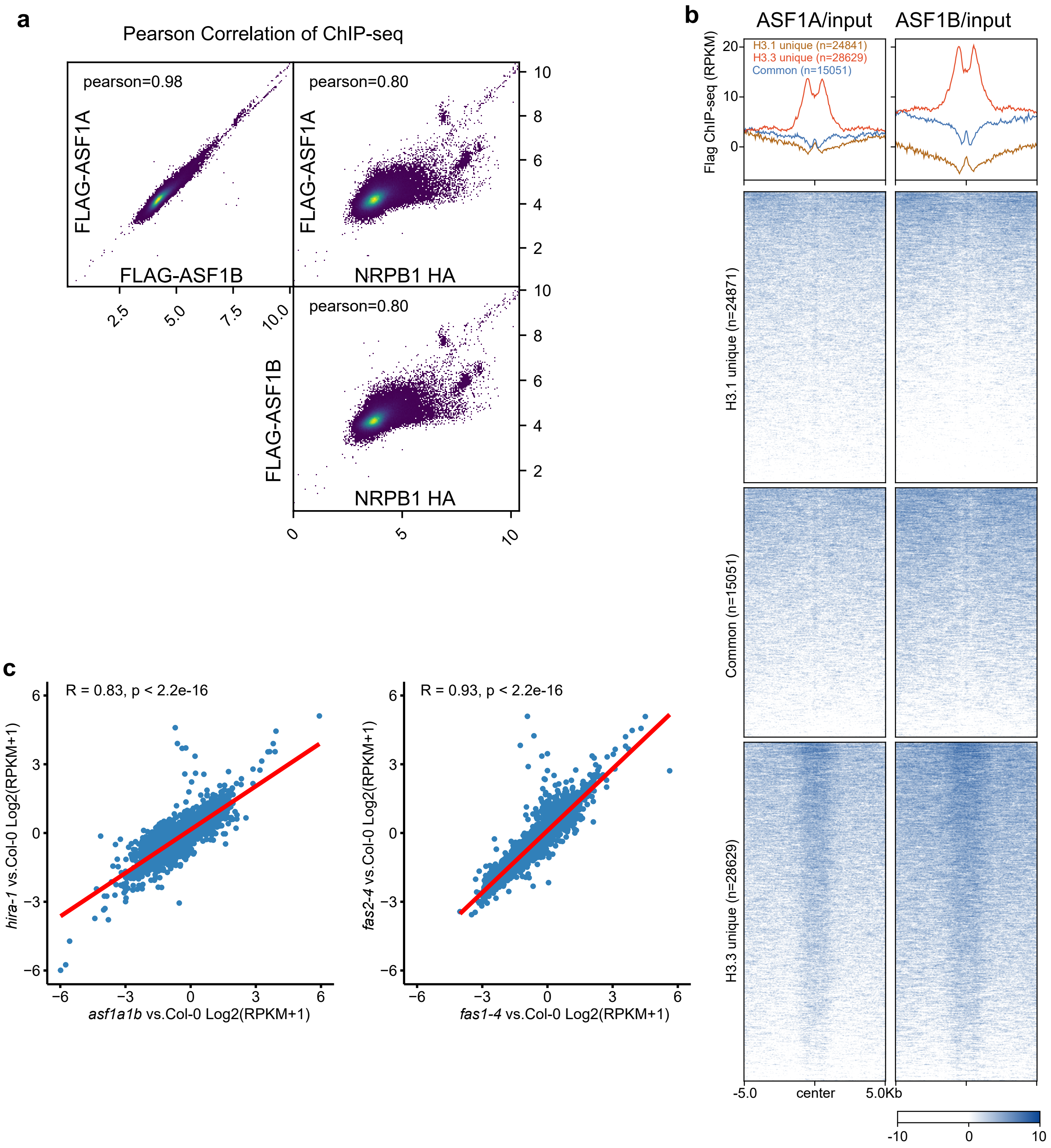

Figure S6. Correlation between ASF1A, ASF1B, NRPB1 and H3.1/H3.3 ChIP-seqs.
(a) Genome-wide pairwise scatterplots for ChIP-seq at 1 Kb bins.
(b) Plotting of ASF1A and B ChIP-seq signal over H3.1 unique (n=24841), H3.3 unique (n=28629), and H3.1/H3.3 common (n=15051) peaks.
(c) Correlation of the expression divergence between H3 chaperone mutants. Graph showing correlation of variation in RNA-seq level of genes (n=3412) that are differentially expressed (FC > 2, FDR < 0.05) in at least one mutant in asf1a1b vs hira-1and fas1-4 vs fas2-4. Pearson method was used to calculate correlation coefficient. R^2 = 0.83 and P-value < 2.2e-16 for *asf1a1b* vs *hira-1*; R^2 = 0.93 and P-value < 2.2e-16 for *fas1-4* vs *fas2-4*.

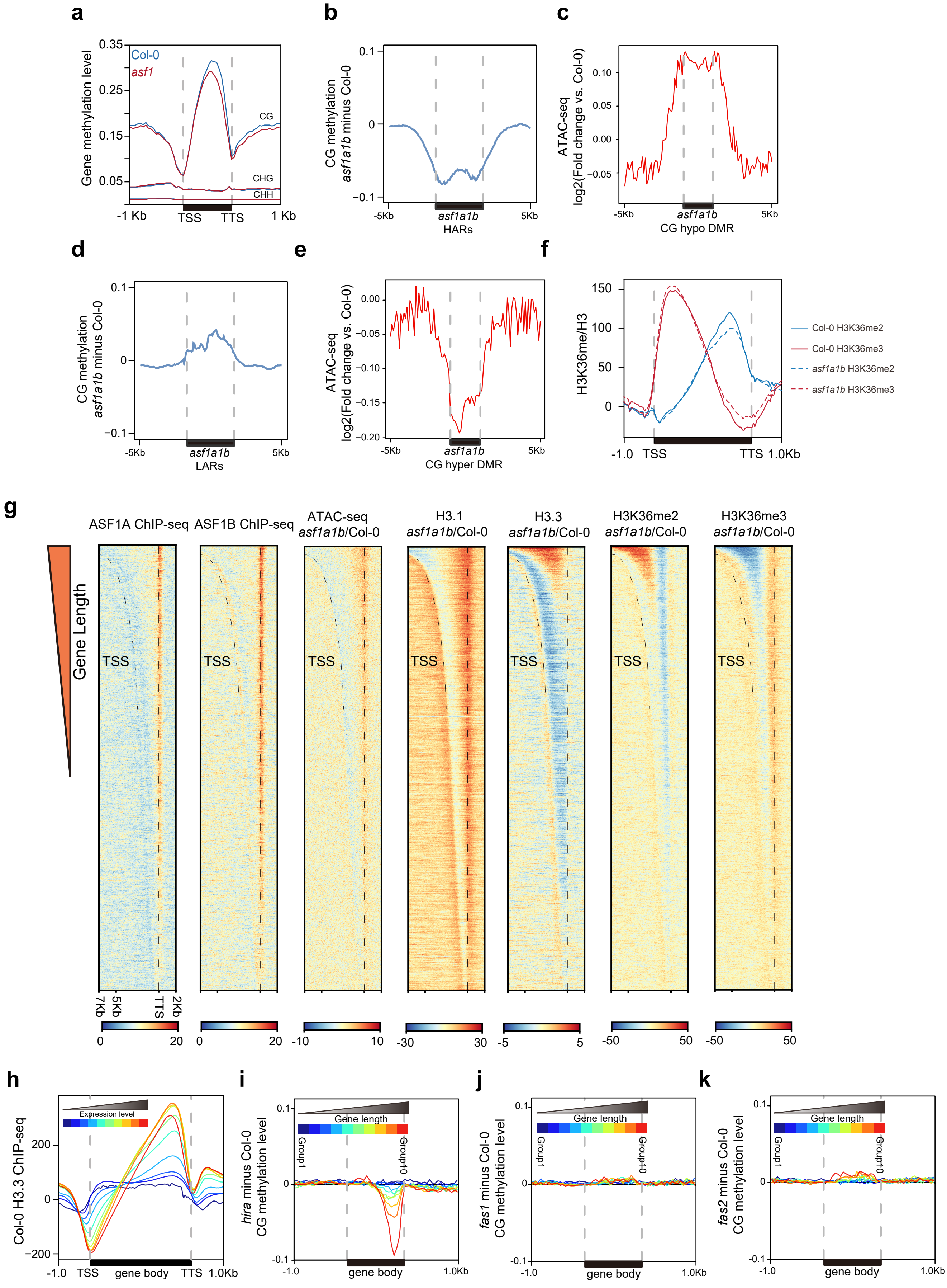

a
Col-0
asf1
Gene methylation level
CG
CHG
CHH
-1 Kb
TSS
TTS
1 Kb
b
CG methylation asf1a1b minus Col-0
asf1a1b
HARs
c
ATAC-seq log2(Fold change vs. Col-0)
asf1a1b
CG hypo DMR
d
CG methylation asf1a1b minus Col-0
asf1a1b
LARs
e
ATAC-seq log2(Fold change vs. Col-0)
asf1a1b
CG hyper DMR
f
H3K36me/H3
Col-0 H3K36me2
Col-0 H3K36me3
asf1a1b H3K36me2
asf1a1b H3K36me3
g
Gene Length
ASF1A ChIP-seq
ASF1B ChIP-seq
ATAC-seq asf1a1b/Col-0
H3.1 asf1a1b/Col-0
H3.3 asf1a1b/Col-0
H3K36me2 asf1a1b/Col-0
H3K36me3 asf1a1b/Col-0
TSS
7Kb
5Kb
TTS
2Kb
h
Col-0 H3.3 ChIP-seq
Expression level
gene body
i
hira minus Col-0 CG methylation level
Gene length
Group1
Group10
j
fas1 minus Col-0 CG methylation level
k
fas2 minus Col-0 CG methylation level

Figure S7. Effects of asf1a1b on the distribution of epigenetic marks.
(a) Metaplot showing DNA methylation level in Col-0 and asf1a1b over the gene body and 1 Kb flanking sequence.
(b) Metaplot showing differential CG methylation level (asf1a1b minus Col-0) over asf1a1b highly accessible regions (HAR, n=1,996).
(c) Metaplot showing differential of chromatin accessibility (asf1a1b/Col-0) over asf1a1b CG hypomethylated DMRs (n=6197).
(d) Metaplot showing differential CG methylation level (asf1a1b minus Col-0) over asf1a1b lowly accessible regions (LAR, n=914).
(e) Metaplot showing differential of chromatin accessibility (asf1a1b/Col-0) over asf1a1b CG hypermethylated DMRs (n=3867).
(f) Metaplot showing H3K36me2/3 level in Col-0 and asf1a1b over the gene body and 1 Kb flanking sequence.
(g) ChIP-seq (ASF1A, ASF1B, H3.1, H3.3, and H3K36me2/3) and ATAC-seq signal over genes ranked by gene length. Top to bottom display genes from long to short.
(h) Metaplot showing H3.3 ChIP-seq signal in Col-0 over genes according to expression level (Group 1 to Group 10 represent gene expression from low to high).
(i-k) Metaplot of CG methylation difference in hira-1 **(i)**, fas1-4 **(j)**, and fas2-4 **(k)** vs. Col-0 over genes grouped by gene length (Group 1 to Group 10 represent gene length from short to long).

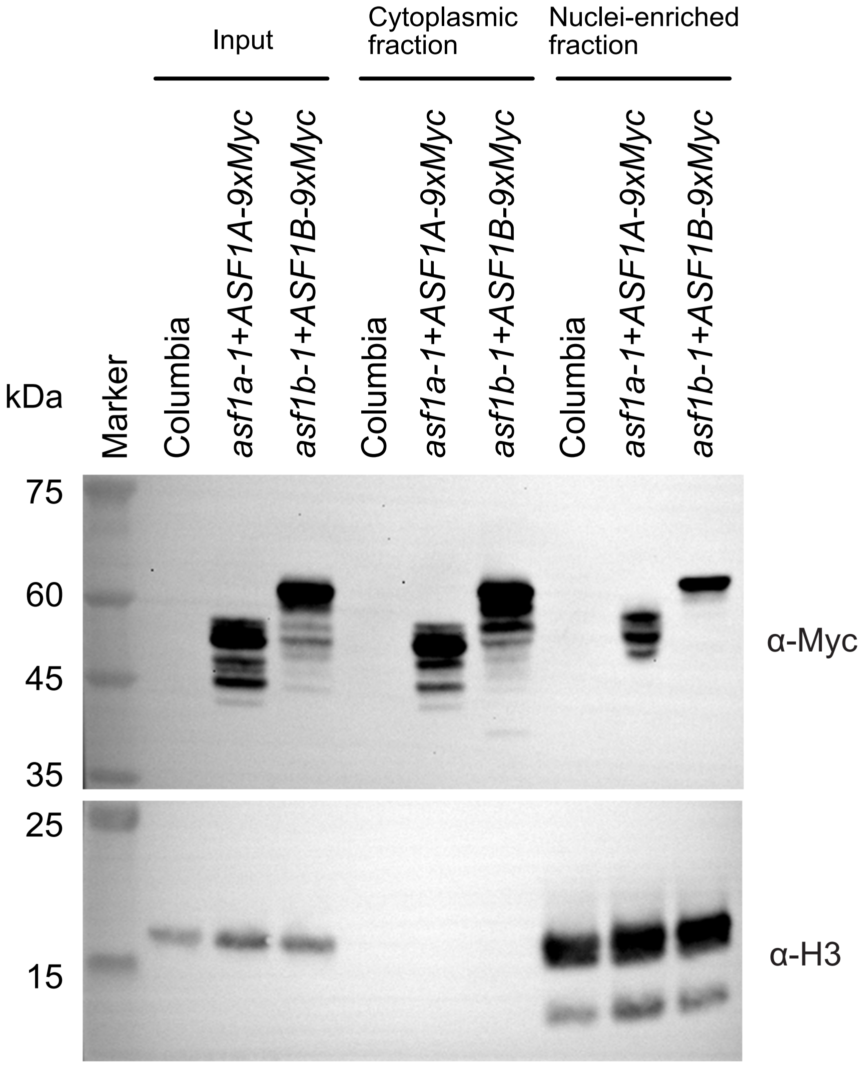

Figure S8. Subcellular fractionation of ASF1 proteins.
Western blot analysis of nuclear and cytoplasmic fractions using an anti-Myc antibody for ASF1A and ASF1B proteins. Western blot using anti-H3 antibody is a control for nuclear proteins. Numbers on the left indicate the protein ladders' molecular weights (kDa). Three experiments were repeated with similar results.

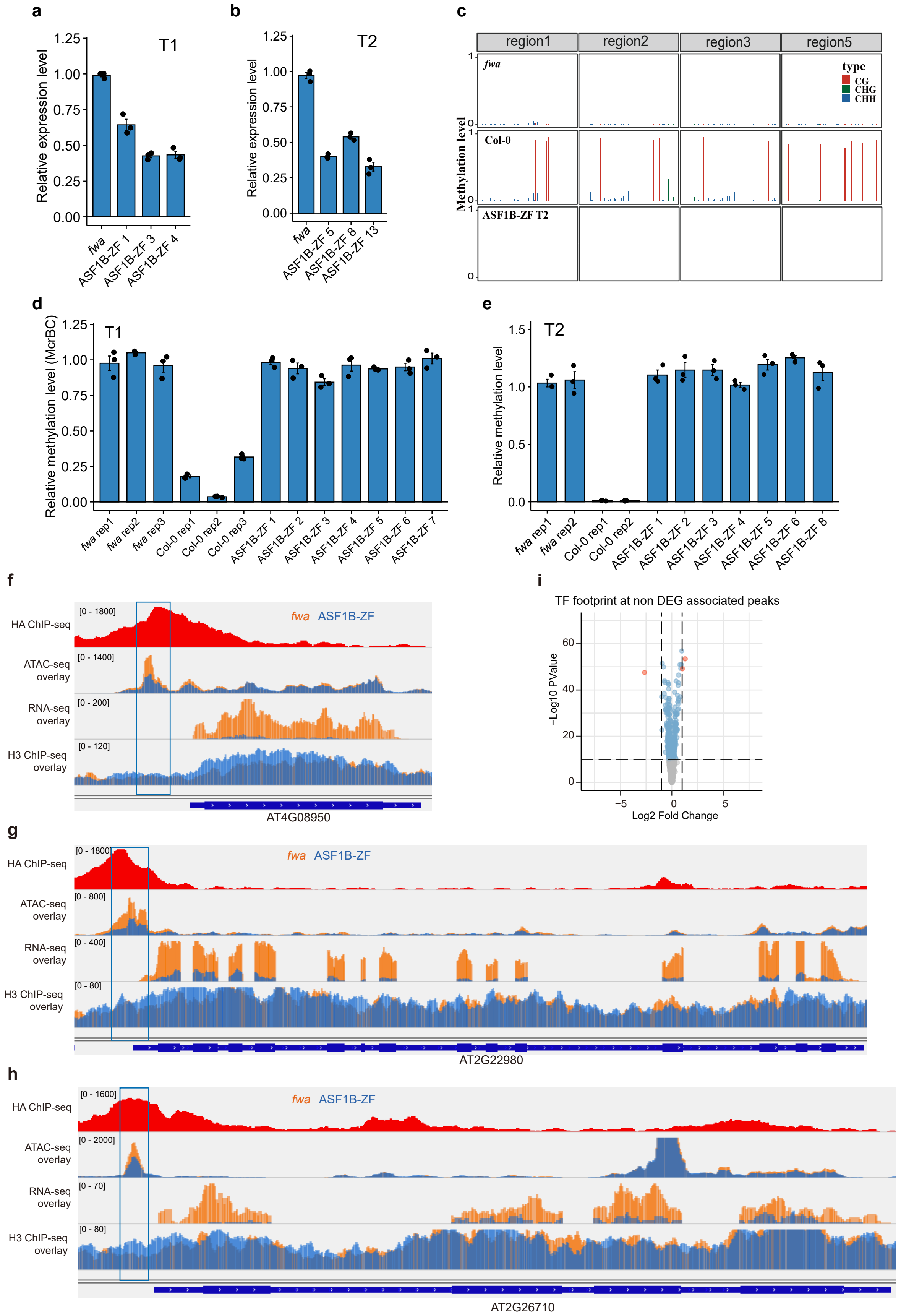

Figure S9. ASF1B-ZF108 effects on FWA expression and TF footprint.

(a-b) The relative expression level of FWA in fwa and ASF1B-ZF108 T1 lines and T2 lines. n=3 biologically independent plants examined. Error bars, mean ± standard error. n=3 technical replicates examined. **(c)** DNA methylation (CG, CHG, and CHH) level over the FWA promoter using bisulfite (BS)-PCR-seq in Col-0 and fwa-4 controls and the ASF1B-ZF108 T2 line. Light red boxes indicate the ASF1B-ZF108 binding sites. **(d-e)** McrBC assay of Col-0 and fwa-4 controls and the ASF1B-ZF108 T1 and T2 lines at the FWA tandem repeat. n=3 biologically independent plants examined. Error bars, mean ± standard error. n=3 biological and technical replicates examined.
(f-h) Screenshot of example genes showing ZF binding assayed by ChIP-seq, chromatin accessibility assayed by ATAC-Seq, and expression level at AT4G08950, AT2G22980, and AT2G26710. Tracks of fwa-4 (orange) and ASF1B-ZF108-3xFlag (blue) are shown overlapped.
(i) Volcano plot showing differential footprint of 572 plant TF motifs distributed at non-DEG associated, down-regulated off-target sites (n=572). P values calculated by two-sided *t* test.

Supplementary Tables

Supplementary Table 1 Identified ASF1-interacting proteins by immunoprecipitation in combination with mass spectrometry (IP-MS) in ASF1A-3xFlag and ASF1B-9xMyc lines. Values indicate spectral counts values for each protein. Corresponding four wild type control for ASF1A and three wild type control for ASF1B did not show significant enrichment of peptides. N/F represents not identified.

ID	Protein	ASF1A				ASF1B		
		Rep1	Rep2	Rep3	Rep4	Rep1	Rep2	Rep3
AT1G66740	ASF1A	84	97	84	92	N/F	N/F	N/F
AT5G38110	ASF1B	N/F	N/F	N/F	N/F	143	122	102
AT4G37210	NASP	71	60	63	33	1	2	2
AT3G44530	HIRA	62	64	126	153	49	212	199
AT4G32820	CABIN1	33	41	116	127	50	107	68
AT5G20930	TOUSLED	18	14	71	47	16	59	51
AT1G21610	UBN1	7	10	30	20	19	56	49
AT1G77310	UBN2	N/F	N/F	23	9	8	12	10

Supplementary Table 2 Segregation ratios of *asf1a-1 asf1b-1 FAS1/fas1-4* and *asf1a-1 asf1b-1 FAS2/fas2-4* genotyped at F3 population.

asf1a1b fas1-4 (het)	**Expected**	**Observed**	***asf1a1b fas2-4 (het)***	**Expected**	**Observed**
FAS1	23.5	48	***FAS2***	18	37
fas1-4	23.5	0	***fas2-4***	18	0
het	47	46	***het***	36	35

Supplementary Table 3 Primers used in this study.

Name	Sequence	Description
ENTR-ASF1AgDNA-F	CCGCGGCCGCCCCCTTCACCTGCTATCAAGCACTTGCAAAGTT	gDNA cloning for ASF1A full length with native promoter
ENTR-ASF1AgDNA-R	GGGTCGGCGCGCCCACCCTTTGATTCCTCAGGTTTTGGTTCTACT	
ENTR-ASF1BgDNA-F	CCGCGGCCGCCCCCTTCACCATGCCACTTACTTATGCTTAAAAAACG	gDNA cloning for ASF1B full length with native promoter
ENTR-ASF1BgDNA-R	GGGTCGGCGCGCCCACCCTTTGTCTCCTGGAGATTTTGTGGCT	
ASF1A-gDNA-GT-F	GATTGATAAAGTTCAGAGGAACATACTT	gDNA genotyping primers for ASF1A
ASF1A-gDNA-GT-R	AGCCTACATAGATAAGCTTCCATTCCA	
ASF1B-gDNA-GT-F	TCATCCTGAGAATGAGCAGACTCT	gDNA genotyping primers for ASF1B
ASF1B-gDNA-GT-R	GGCTTACCAGAATGTGATCATG	
H3.1-flag/myc-F	GTGAACGAAGATGATGAAGATTAA	Cloning primers for H3.1 (HTR13)
H3.1-flag/myc -R	AGCTCTTTCTCCTCTGATTCTCCT	
H3.3-flag/myc -F	TTACGATTTAGATCCGATATAACA	Cloning primers for H3.3 (HTR5)
H3.3-flag/myc -R	AGCACGTTCTCCTCTGATCCTGCG	
FWA_McRBc-F	TTGGGTTTAGTGTTTACTTG	McRBc primers for FWA promoter epiallele region
FWA_McRBc-R	GAATGTTGAATGGGATAAGGTA	
FWA_qRT-PCR-F	GTATGAGTTGCTTCTCCAGCAAAG	qRT PCR primers for FWA promoter epiallele region
FWA_qRT-PCR-R	GAGGATGGCTGCAACAAGTGT	
FWA_bsPCR_region1-F	TCATATAAAAAAAAAATTAAATTTCATTTCACAATAACCATT	BS-PCR primer for FWA region1
FWA_bsPCR_region1-R	GTATGGGYTTYGATAAAGAATATATGAGATTYT	
FWA_bsPCR_region2-F	CTCATATATACCTTATCCCATTCAACATTCATA	BS-PCR primer for FWA region2
FWA_bsPCR_region2-R	AAGATYTGATATTTGGYTGGAAAAAAYAATAATAAT	
FWA_bsPCR_region3-F	CRCTCTTTATCCCATTCAACATTCATAC	BS-PCR primer for FWA region3
FWA_bsPCR_region3-R	TTTGGTTGAAAAAAATAATAAAAATTTGATTGTYAGTAT	
FWA_bsPCR_region5-F	AAGYTGTATTTTTGTGGAAGATAGTATTATTTAAAAATTA	BS-PCR primer for FWA region5
FWA_bsPCR_region5-R	CAAACAATTTTAATCATCAAAAAAAAACTRAATTCCT	

Supplementary Table 4 Published ChIP-seq data used in this study.

Data	SRA link
H3K27me3	SRR10905142[1]
H3K27ac	SRR1509479[2]
H3K9me2	SRR6410845[3]
H3K4me3	SRR10905140[1]
H3Ac	SRR5837284[4]
H3K9Ac	SRR4733909[5]
H4K16ac	SRR6364625[6]
H1	SRR5364423[7]
H2A.Z	SRR8695714[8]

References

1 Liu, Q. *et al.* The characterization of Mediator 12 and 13 as conditional positive gene regulators in Arabidopsis. *Nature communications* **11**, 1-13 (2020).

2 Zhu, B., Zhang, W., Zhang, T., Liu, B. & Jiang, J. Genome-wide prediction and validation of intergenic enhancers in Arabidopsis using open chromatin signatures. *The Plant cell* **27**, 2415-2426 (2015).

3 Harris, C. J. *et al.* A DNA methylation reader complex that enhances gene transcription. *Science* **362**, 1182-1186 (2018).

4 Jin, H. *et al.* Salicylic acid-induced transcriptional reprogramming by the HAC–NPR1–TGA histone acetyltransferase complex in Arabidopsis. *Nucleic acids research* **46**, 11712-11725 (2018).

5 Jégu, T. *et al.* The Arabidopsis SWI/SNF protein BAF60 mediates seedling growth control by modulating DNA accessibility. *Genome biology* **18**, 114 (2017).

6 Chen, X. *et al.* Canonical and noncanonical actions of Arabidopsis histone deacetylases in ribosomal RNA processing. *The Plant cell* **30**, 134-152 (2018).

7 Wollmann, H. *et al.* The histone H3 variant H3. 3 regulates gene body DNA methylation in Arabidopsis thaliana. *Genome biology* **18**, 94 (2017).

8 Wang, Y. *et al.* NAP1-RELATED PROTEIN1 and 2 negatively regulate H2A. Z abundance in chromatin in Arabidopsis. *Nature communications* **11**, 1-10 (2020).
