# Supplementary material for: Histone chaperone ASF1 mediates H3.3-H4 deposition in Arabidopsis
Source: Nat Commun. 2022 Nov 15;13:6970. doi: 10.1038/s41467-022-34648-0 (PMC9666630; doi:10.1038/s41467-022-34648-0)
Supplement: Supplementary file 3 — Reporting Summary [file 41467_2022_34648_MOESM3_ESM.pdf]

nature portfolio

Corresponding author(s): Steven E. Jacobsen, Israel Ausin

Last updated by author(s): Oct 24, 2022

Reporting Summary

Nature Portfolio wishes to improve the reproducibility of the work that we publish. This form provides structure for consistency and transparency in reporting. For further information on Nature Portfolio policies, see our Editorial Policies and the Editorial Policy Checklist.

Statistics

For all statistical analyses, confirm that the following items are present in the figure legend, table legend, main text, or Methods section.

n/a	Confirmed	
☐	☒	The exact sample size (*n*) for each experimental group/condition, given as a discrete number and unit of measurement
☐	☒	A statement on whether measurements were taken from distinct samples or whether the same sample was measured repeatedly
☐	☒	The statistical test(s) used AND whether they are one- or two-sided<br>*Only common tests should be described solely by name; describe more complex techniques in the Methods section.*
☒	☐	A description of all covariates tested
☐	☒	A description of any assumptions or corrections, such as tests of normality and adjustment for multiple comparisons
☐	☒	A full description of the statistical parameters including central tendency (e.g. means) or other basic estimates (e.g. regression coefficient) AND variation (e.g. standard deviation) or associated estimates of uncertainty (e.g. confidence intervals)
☐	☒	For null hypothesis testing, the test statistic (e.g. *F*, *t*, *r*) with confidence intervals, effect sizes, degrees of freedom and *P* value noted<br>*Give P values as exact values whenever suitable.*
☒	☐	For Bayesian analysis, information on the choice of priors and Markov chain Monte Carlo settings
☐	☒	For hierarchical and complex designs, identification of the appropriate level for tests and full reporting of outcomes
☒	☐	Estimates of effect sizes (e.g. Cohen's *d*, Pearson's *r*), indicating how they were calculated

Our web collection on statistics for biologists contains articles on many of the points above.

Software and code

Policy information about availability of computer code

Data collection	no software was used for data collection.
Data analysis	ATAC-seq analysis<br>ATAC-seq reads adaptors were removed with trim_galore. The reads were then mapped to Arabidopsis thaliana reference genome TAIR10 using Bowtie2 (-X 2000 -m 1). Reads of chloroplast and mitochondrial DNA were filtered and duplicate reads were removed using Samtools . ATAC-Seq open chromatin peaks of each replicate were called using MACS2 with parameters -p 0.01 --nomodel --shift -100 --extsize 200. Consensus set of chromatin peaks of each samples were merged by bedtools (v2.26.0) intersect with allowing 10 base pairs distance. edgeR was used to define significantly changed peaks [Fold Change, (FC) >2 and False Discovery Rate, (FDR)<0.05]. Chromosomal distributions of ATAC-seq were calculated by dividing Arabidopsis chromosomes into 100 Kb sized bins and count reads at each bin with bedtools. ATAC-seq peak distribution was annotated with ChIPseeker. Arabidopsis protein coding genes were ranked and divided into 10 quantiles according to gene coding region length, and coding region of each gene was divided in proportion into 10 quantiles. Transcriptional factor footprints were analyzed by TOBIAS with 572 plant TF motifs downloaded from Jasper (http://jaspar.genereg.net/).<br><br>RNA-seq analysis<br>Cleaned short reads were aligned to reference genome tair10 by Bowtie2 (v2.1.0), and expression abundance was calculated by RSEM with default parameters. Heatmaps were visualized with the R package pheatmap. To reduce false positive of differential expression, transcripts with less than 5 reads of all replicates in total were regarded as lowly expressed genes and have been removed in subsequent analysis. Differential expression analysis was conducted using edgeR. A threshold of p value < 0.05 and Fold Change > 2 were used to decide whether significant expression difference exists between samples.<br><br>ChIP-seq analysis<br>ChIP-seq fastq reads were aligned to the TAIR10 reference genome with Bowtie (v1.1.2), allowing only uniquely mapping reads with 0 mismatches. Duplicated reads were removed by Samtools. ChIP-seq peaks were called by MACS2 (v2.1.1) and annotated with ChIPseeker.

Differential peaks were called by bdgdiff function in MACS2. ChIP-seq data metaplots were plotted by deeptools (v2.5.1). For Pol II 5' occupancy analysis, Pol II occupancy was calculated based normalized reads count (RPKM) on a TSS +/- 200 bp region and a TSS +500 bp to TTS gene body region by bedtools. Detailed information for published ChIP-seq datasets is listed in Supplementary Table 2.

Whole genome bisulfite sequencing (BS-seq) analysis
Trim_galore (http://www.bioinformatics.babraham.ac.uk/projects/trim_galore/) was used to trim adapters after filtering low quality reads. BS-seq reads were aligned to TAIR10 reference genome by Bismark (v0.18.2) with default settings. Reads with three or more consecutive CHH sites were considered as unconverted reads and filtered. DNA methylation levels were defined as #C/ (#C + #T). DMRs (Differentially Methylated Regions) were called by DMRcaller with p < 0.01 for where the differences in CG, CHG, and CHH methylation were at least 0.4, 0.2, and 0.1, respectively.

TSS-seq analysis
TSS-seq reads were trimmed with Trim_galore and 5' end UMI barcodes were trimmed using UMI-Tools. The reads were then aligned to TAIR10 genome assembly using STAR (v2.7.0e). Mapping files were filtered with MAPQ<10 and deduplicated using SAMtools (v1.9) before converted to stranded Bedgraph files using bedtools (v2.26.0). TSS peaks were identified with CAGEfightR (v0.99.0) and differential TSS peaks [Fold Change, (FC) >2 and p value<0.05] were called with DESeq2 (v1.28.1).

BS-PCR analysis
BS-PCR data were trimmed with primer sequences and mapped to TAIR10 reference genome with bsmap (v2.90) allowing 2 mismatches and 1 best hit (-v 2 -w 1).

For manuscripts utilizing custom algorithms or software that are central to the research but not yet described in published literature, software must be made available to editors and reviewers. We strongly encourage code deposition in a community repository (e.g. GitHub). See the Nature Portfolio guidelines for submitting code & software for further information.

Data

Policy information about availability of data

All manuscripts must include a data availability statement. This statement should provide the following information, where applicable:
- Accession codes, unique identifiers, or web links for publicly available datasets
- A description of any restrictions on data availability
- For clinical datasets or third party data, please ensure that the statement adheres to our policy

All high-throughput sequencing data generated in this study are accessible at NCBI's Gene Expression Omnibus (GEO) via GEO Series accession number GSE188493 (https://www.ncbi.nlm.nih.gov/geo/query/acc.cgi?acc=GSE188493).

IP-MS data is available at PRIDE database with project accession: PXD035578

Field-specific reporting

Please select the one below that is the best fit for your research. If you are not sure, read the appropriate sections before making your selection.

☒ Life sciences ☐ Behavioural & social sciences ☐ Ecological, evolutionary & environmental sciences

For a reference copy of the document with all sections, see nature.com/documents/nr-reporting-summary-flat.pdf

Life sciences study design

All studies must disclose on these points even when the disclosure is negative.

Sample size	No sample size calculation was performed.
Data exclusions	No data exclusion in the study.
Replication	Two replicates for ChIP-seq. Two replicates for ATAC-seq. Three replicates for RNA-seq samples. Two replicates for WGBS data. Three replicates for TSS-seq data. Two replicates for BS-PCR.
Randomization	For all experiments, treatment and control samples were grown side by side, each replicate on separate plate.
Blinding	No blinding used.

Reporting for specific materials, systems and methods

We require information from authors about some types of materials, experimental systems and methods used in many studies. Here, indicate whether each material, system or method listed is relevant to your study. If you are not sure if a list item applies to your research, read the appropriate section before selecting a response.

Materials & experimental systems

n/a	Involved in the study
☐	☒ Antibodies
☒	☐ Eukaryotic cell lines
☒	☐ Palaeontology and archaeology
☒	☐ Animals and other organisms
☒	☐ Human research participants
☒	☐ Clinical data
☒	☐ Dual use research of concern

Methods

n/a	Involved in the study
☐	☒ ChIP-seq
☒	☐ Flow cytometry
☒	☐ MRI-based neuroimaging

Antibodies

Antibodies used

anti-FLAG M2 (Sigma)
anti-HA (Roche)
anti-Pol II (Ab26721, Abcam)
anti-H3 (Ab1791, Abcam)
anti-H3K36me2 (Ab9049, Abcam)
anti-H3K36me3 (Ab9050, Abcam)
Anti-NRPB1 antibody was raised in rabbits and further affinity-purified by ABClonal (China) using the peptide HEGDKKDKTGKKDASKDDK.
anti-Myc (Cat# 2278, Cell Signaling Technology)

Validation

anti-FLAG M2 (Sigma): https://www.sigmaaldrich.com/catalog/product/sigma/f1804
anti-HA11867423001 (Roche): https://www.sigmaaldrich.com/catalog/product/roche/roahaha?lang=en&region=US
anti-Pol II (Ab26721, Abcam): https://www.abcam.com/rna-polymerase-ii-ctd-repeat-ysptsps-antibody-chip-grade-ab26721.html
anti-H3 (Ab1791, Abcam): https://www.abcam.com/histone-h3-antibody-nuclear-marker-and-chip-grade-ab1791.html
anti-H3K36me2 (Ab9049, Abcam): https://www.abcam.com/histone-h3-di-methyl-k36-antibody-chip-grade-ab9049.html
anti-H3K36me3 (Ab9050, Abcam): https://www.abcam.com/histone-h3-tri-methyl-k36-antibody-chip-grade-ab9050.html
anti-Myc: https://www.cellsignal.com/products/primary-antibodies/myc-tag-71d10-rabbit-mab/2278
Anti-NRPB1 antibody was validated by Manufacturer and data provided in this paper.

ChIP-seq

Data deposition

☒ Confirm that both raw and final processed data have been deposited in a public database such as GEO.

☒ Confirm that you have deposited or provided access to graph files (e.g. BED files) for the called peaks.

Data access links
May remain private before publication.

All high-throughput sequencing data generated in this study are accessible at NCBI's Gene Expression Omnibus (GEO) via GEO Series accession number GSE188493 (https://www.ncbi.nlm.nih.gov/geo/query/acc.cgi?acc=GSE188493). Enter token ynofsissxjqxhup into the box.

Files in database submission

ChIP-seq_data/ASF1_flag/repeat1:
ASF1a_peaks_peaks.narrowPeak
ASF1a_peaks_peaks.xls
ASF1a_peaks_summits.bed
ASF1b_peaks_peaks.narrowPeak
ASF1b_peaks_peaks.xls
ASF1b_peaks_summits.bed
FALG_ASF1B_rep1.bw
FALG_ASF1B_rep1_1.fastq.gz
FALG_ASF1B_rep1_2.fastq.gz
FLAG_ASF1A_rep1.bw
FLAG_ASF1A_rep1_1.fastq.gz
FLAG_ASF1A_rep1_2.fastq.gz
FLAG_WT.bw
FLAG_WT_1.fastq.gz
FLAG_WT_2.fastq.gz

ChIP-seq_data/ASF1_flag/repeat2:
FLAG-ASF1A_rep2.bw
FLAG-ASF1A_rep2_1.fastq.gz
FLAG-ASF1A_rep2_2.fastq.gz
FLAG-ASF1B_rep2.bw

FLAG-ASF1B_rep2_1.fastq.gz
FLAG-ASF1B_rep2_2.fastq.gz
FLAG-H3-1.bw
FLAG-H3-3.bw
FLAG-WT.bw
FLAG-WT2.bw
FLAG-WT2_1.fastq.gz
FLAG-WT2_2.fastq.gz
FLAG-WT_1.fastq.gz
FLAG-WT_2.fastq.gz

ChIP-seq_data/H3.1_H3.3:
ChIP-3-1-WT.bw
ChIP-3-1-WT_R1.fastq.gz
ChIP-3-1-WT_R2.fastq.gz
ChIP-3-1-asf.bw
ChIP-3-1-asf_R1.fastq.gz
ChIP-3-1-asf_R2.fastq.gz
ChIP-3-3-WT.bw
ChIP-3-3-WT_R1.fastq.gz
ChIP-3-3-WT_R2.fastq.gz
ChIP-3-3-asf.bw
ChIP-3-3-asf_R1.fastq.gz
ChIP-3-3-asf_R2.fastq.gz
ChIP-WT.bw
ChIP-WT_R1.fastq.gz
ChIP-WT_R2.fastq.gz

ChIP-seq_data/H3K36/repeat1:
Col_H3K36di_1.fastq.gz
Col_H3K36di_peaks_peaks.narrowPeak
Col_H3K36di_peaks_peaks.xls
Col_H3K36di_peaks_summits.bed
Col_H3K36tri_1.fastq.gz
Col_H3K36tri_peaks_peaks.narrowPeak
Col_H3K36tri_peaks_peaks.xls
Col_H3K36tri_peaks_summits.bed
Col_H3_1.fastq.gz
asf1_H3K36di_1.fastq.gz
asf1_H3K36di_peaks_peaks.narrowPeak
asf1_H3K36di_peaks_peaks.xls
asf1_H3K36di_peaks_summits.bed
asf1_H3K36tri_1.fastq.gz
asf1_H3K36tri_peaks_peaks.narrowPeak
asf1_H3K36tri_peaks_peaks.xls
asf1_H3K36tri_peaks_summits.bed
asf1_H3_1.fastq.gz

ChIP-seq_data/H3K36/repeat2:
WT2-H3K36me2_rep2_1.fastq.gz
WT2-H3K36me2_rep2_2.fastq.gz
WT2-H3K36me3_rep2_1.fastq.gz
WT2-H3K36me3_rep2_2.fastq.gz
WT2-H3_rep2_1.fastq.gz
WT2-H3_rep2_2.fastq.gz
WT2_H3.bw
WT2_H3K36me2.bw
WT2_H3K36me2_peaks.narrowPeak
WT2_H3K36me2_peaks.xls
WT2_H3K36me2_summits.bed
WT2_H3K36me2_vs_h3.bw
WT2_H3K36me3.bw
WT2_H3K36me3_peaks.narrowPeak
WT2_H3K36me3_peaks.xls
WT2_H3K36me3_summits.bed
WT2_H3K36me3_vs_h3.bw
asf1-H3K36me2_rep2_1.fastq.gz
asf1-H3K36me2_rep2_2.fastq.gz

asf1-H3K36me3_rep2_1.fastq.gz
asf1-H3K36me3_rep2_2.fastq.gz
asf1-H3_rep2_1.fastq.gz
asf1-H3_rep2_2.fastq.gz
asf1_H3K36me2_peaks.narrowPeak
asf1_H3K36me2_peaks.xls
asf1_H3K36me2_summits.bed
asf1_H3K36me3_peaks.narrowPeak
asf1_H3K36me3_peaks.xls
asf1_H3K36me3_summits.bed
asf1a_H3.bw
asf1a_H3K36me2.bw
asf1a_H3K36me2_vs_h3.bw
asf1a_H3K36me3.bw
asf1a_H3K36me3_vs_h3.bw

ChIP-seq_data/NRPB1:
Col1_NRPB1_ChIP_rep1_D1.bw
Col1_NRPB1_ChIP_rep1_D1.fastq.gz
Col1_NRPB1_ChIP_rep2_D3.bw
Col1_NRPB1_ChIP_rep2_D3.fastq.gz

ChIP-seq_data/Pol2/repeat1:
asf1_ASF_INPUT.bw
asf1_ASF_INPUT.fq.gz
asf1_CT.bw
asf1_h3.bw
asf1_h3.fq.gz
asf1_h3_vs_CT_B_peaks.narrowPeak
asf1_h3_vs_CT_B_peaks.xls
asf1_h3_vs_CT_B_summits.bed
asf1_pol2_new.bw
asf1_pol2_new.fq.gz
asf1_pol2_new_vs_CT_B_peaks.narrowPeak
asf1_pol2_new_vs_CT_B_peaks.xls
asf1_pol2_new_vs_CT_B_summits.bed
col_ASF_INPUT.bw
col_ASF_INPUT.fq.gz
col_CT.bw
col_CT.fq.gz
col_h3.bw
col_h3.fq.gz
col_h3_vs_CT_B_peaks.narrowPeak
col_h3_vs_CT_B_peaks.xls
col_h3_vs_CT_B_summits.bed
col_pol2_new.bw
col_pol2_new.fq.gz
col_pol2_new_vs_CT_B_peaks.narrowPeak
col_pol2_new_vs_CT_B_peaks.xls
col_pol2_new_vs_CT_B_summits.bed

ChIP-seq_data/Pol2/repeat2:
Col_h3k4me3_peaks_peaks.narrowPeak
Col_h3k4me3_peaks_peaks.xls
Col_h3k4me3_peaks_summits.bed
ZCol_h3.bw
ZCol_h3.fq.gz
ZCol_h3k4me3.bw
ZCol_h3k4me3.fq.gz
ZCol_pol2.bw
ZCol_pol2.fq.gz
Zas1_h3k4me3.bw
Zas1_h3k4me3.fq.gz
Zasf1_h3.bw
Zasf1_h3.fq.gz
Zasf1_pol2.bw
Zasf1_pol2.fq.gz
asf1_h3k4me3_peaks_peaks.narrowPeak

asf1_h3k4me3_peaks_peaks.xls
asf1_h3k4me3_peaks_summits.bed

ZF-ASF1B/ChIP-seq:
H3-ASF1B_rep1.bw
H3-ASF1B_rep1_1.fastq.gz
H3-ASF1B_rep1_2.fastq.gz
H3-ASF1B_rep2.bw
H3-ASF1B_rep2_1.fastq.gz
H3-ASF1B_rep2_2.fastq.gz
H3-fwa1.bw
H3-fwa1_1.fastq.gz
H3-fwa1_2.fastq.gz
H3-fwa2.bw
H3-fwa2_1.fastq.gz
H3-fwa2_2.fastq.gz
ZF-ASF1B-H3_rep1_peaks_peaks.narrowPeak
ZF-ASF1B-H3_rep2_peaks_peaks.narrowPeak
fwa-H3_rep1_peaks_peaks.narrowPeak
fwa-H3_rep2_peaks_peaks.narrowPeak
input-ASF1B_rep1.bw
input-ASF1B_rep1_1.fastq.gz
input-ASF1B_rep1_2.fastq.gz
input-ASF1B_rep2.bw
input-ASF1B_rep2_1.fastq.gz
input-ASF1B_rep2_2.fastq.gz
input-fwa1.bw
input-fwa1_1.fastq.gz
input-fwa1_2.fastq.gz
input-fwa2.bw
input-fwa2_1.fastq.gz
input-fwa2_2.fastq.gz

H3-1-WT_R1.fastq.gz
H3-3-WT_R1.fastq.gz
H3-1-asf_R1.fastq.gz
H3-3-asf_R1.fastq.gz
WT_R1.fastq.gz
myc-H3-1-ASF1B-ZF_S17_L002_R1_001.fastq.gz
myc-H3-3-ASF1B-ZF_S18_L002_R1_001.fastq.gz
myc-H3-1-fwa_S15_L002_R1_001.fastq.gz
myc-H3-3-fwa_S16_L002_R1_001.fastq.gz
myc-fwa_S19_L002_R1_001.fastq.gz
input-H3-1-ASF1B-ZF_S22_L002_R1_001.fastq.gz
input-H3-3-ASF1B-ZF_S23_L002_R1_001.fastq.gz
input-H3-1-fwa_S20_L002_R1_001.fastq.gz
input-H3-3-fwa_S21_L002_R1_001.fastq.gz
input-fwa_S24_L002_R1_001.fastq.gz
H3-1-WT_R2.fastq.gz
H3-3-WT_R2.fastq.gz
H3-1-asf_R2.fastq.gz
H3-3-asf_R2.fastq.gz
WT_R2.fastq.gz
myc-H3-1-ASF1B-ZF_S17_L002_R2_001.fastq.gz
myc-H3-3-ASF1B-ZF_S18_L002_R2_001.fastq.gz
myc-H3-1-fwa_S15_L002_R2_001.fastq.gz
myc-H3-3-fwa_S16_L002_R2_001.fastq.gz
myc-fwa_S19_L002_R2_001.fastq.gz
input-H3-1-ASF1B-ZF_S22_L002_R2_001.fastq.gz
input-H3-3-ASF1B-ZF_S23_L002_R2_001.fastq.gz
input-H3-1-fwa_S20_L002_R2_001.fastq.gz
input-H3-3-fwa_S21_L002_R2_001.fastq.gz
input-fwa_S24_L002_R2_001.fastq.gz

H3-1-WT_rep2.bw
H3-3-WT_rep2.bw
H3-1-asf_rep2.bw
H3-3-asf_rep2.bw

WT_rep2.bw
myc-H3-1-fwa_S15_L002.bw
myc-H3-3-fwa_S16_L002.bw
myc-H3-1-ASF1B-ZF_S17_L002.bw
myc-H3-3-ASF1B-ZF_S18_L002.bw
myc-fwa_S19_L002.bw
input-H3-1-fwa_S20_L002.bw
input-H3-3-fwa_S21_L002.bw
input-H3-1-ASF1B-ZF_S22_L002.bw
input-H3-3-ASF1B-ZF_S23_L002.bw
input-fwa_S24_L002.bw
WT_H3.1_peaks_rep2.narrowPeak
WT_H3.3_peaks_rep2.narrowPeak
asf1_H3.1_peaks_rep2.narrowPeak
asf1_H3.3_peaks_rep2.narrowPeak
H3.1-fwa_peaks_peaks.narrowPeak
H3.3-fwa_peaks_peaks.narrowPeak
H3.1-ASF1B-ZF_peaks_peaks.narrowPeak
H3.3-ASF1B-ZF_peaks_peaks.narrowPeak
myc-fwa_peaks_peaks.narrowPeak

Genome browser session
(e.g. UCSC)

Provide a link to an anonymized genome browser session for "Initial submission" and "Revised version" documents only, to enable peer review. Write "no longer applicable" for "Final submission" documents.

Methodology

Replicates

For H3 ChIP-Seq experiment, two replicates were performed, along with DNA input control.
For Pol II ChIP-Seq experiment, two replicates were performed, along with DNA input control.
For H3K36me2 ChIP-Seq experiment, two replicates were performed, along with H3 input control.
For H3K36me3 ChIP-Seq experiment, two replicates were performed, along with H3 input control.
For anti-NRPB1 ChIP-Seq experiment, single replicate was performed.
For ASF1A ChIP-Seq experiment, two replicates were performed, along with flag control.
For ASF1B ChIP-Seq experiment, two replicates were performed, along with flag control.
For H3.1-flag ChIP-Seq experiment in Col-0 background, single replicate was performed, along with flag control.
For H3.3-flag ChIP-Seq experiment in Col-0 background, single replicate was performed, along with flag control.
For H3.1-flag ChIP-Seq experiment in asf1a1b mutant background, single replicate was performed, along with flag control.
For H3.3-flag ChIP-Seq experiment in asf1a1b mutant background, single replicate was performed, along with flag control.

Sequencing depth

sample; Total number of reads; Uniquely mapped reads; length of reads; SE/PE
ChIP-WT; 23434482; 18502238; 50; PE
ChIP-3-1-WT; 48625114; 41675010; 50; PE
ChIP-3-3-WT; 74767018; 65954970; 50; PE
ChIP-3-1-asf1a1b; 40757426; 33099130; 50; PE
ChIP-3-3-asf1a1b; 70699497; 62227180; 50; PE
FLAG-ASF1A_rep2; 47445979; 42735852; 50; PE
FLAG-ASF1B_rep2; 46594725; 41289021; 50; PE
FLAG-WT_rep2; 19697530; 16667812; 50; PE
FLAG-ASF1A_rep1; 11219614; 10243510; 50; PE
FLAG-ASF1B_rep1; 7695634; 6343596; 50; PE
FLAG-WT_rep1; 7784981; 5078101; 50; PE
Col-0_H3_rep1; 49227082; 34164638; 50; SE
Col-0_H3K36me2_rep1; 20942660; 18680115; 50; SE
Col-0_H3K36me3_rep1; 34809602; 27882068; 50; SE
asf1_H3_rep1; 45877979; 31874615; 50; SE
asf1_H3K36me2_rep1; 23720106; 21249271; 50; SE
asf1_H3K36me3_rep1; 36298678; 33395643; 50; SE
Col-0_H3_rep2; 64384218; 40483490; 50; PE
Col-0_H3K36me2_rep2; 39344996; 31360433; 50; PE
Col-0_H3K36me3_rep2; 54341652; 46094477; 50; PE
asf1_H3_rep2; 64551068; 41552312; 50; PE
asf1_H3K36me2_rep2; 51735492; 39972383; 50; PE
asf1_H3K36me3_rep2; 54217424; 45811110; 50; PE
Col-0_Pol2_rep1; 75633817; 73599159; 50; SE
Col-0_input_rep1; 55927309; 47460657; 50; SE
asf1_Pol2_rep1; 89641244; 85449111; 50; SE

asf1_input_rep1; 75162991; 63020421; 50; SE
Col-0_Pol2_rep2; 11570183; 11075756; 50; SE
Col-0_input_rep2; 12486652; 6488268; 50; SE
asf1_Pol2_rep2; 15700515; 14961075; 50; SE
asf1_input_rep2; ; ; 50; SE
Col_NRPB1_rep1; 13176147; 7484375; 50; SE
Col_NRPB1_rep2; 11208896; 10169371; 50; SE
fwa_H3_rep1; 44371020; 39460776; 50; PE
fwa_input_rep1; 22124502; 20242150; 50; PE
ASF1B_ZF_H3_rep1; 46293877; 41381486; 50; PE
ASF1B_ZF_input_rep1; 21783098; 19887207; 50; PE
fwa_H3_rep2; 31102349; 27416087; 50; PE
fwa_input_rep2; 21486786; 19655196; 50; PE
ASF1B_ZF_H3_rep2; 36480350; 33059454; 50; PE
ASF1B_ZF_input_rep2; 26104089; 24298018; 50; PE

Antibodies	anti-FLAG M2 (Sigma)<br>anti-HA (Roche)<br>anti-Pol II (Ab26721, Abcam)<br>anti-H3 (Ab1791, Abcam)<br>anti-H3K36me2 (Ab9049, Abcam)<br>anti-H3K36me3 (Ab9050, Abcam)<br>Anti-NRPB1 antibody was raised in rabbits and further affinity-purified by ABClonal (China) using the peptide HEGDKKDKTGKKDASKDDK.
Peak calling parameters	MACS2: '-f BAM -g 1.3e+8 -q 0.05 --extsize 147'
Data quality	All identified peaks in the study were called with a qval threshold of 0.01 (FDR 1%).
Software	Bowtie (v1.1.2),<br>Samtools (v1.9)<br>MACS2 (v2.1.1)<br>ChIPseeker<br>deeptools (v2.5.1).<br>bedtools (v2.26.0)
